# Impact of intracellular hemin on N-type inactivation of voltage-gated K^+^ channels

**DOI:** 10.1007/s00424-020-02386-1

**Published:** 2020-05-10

**Authors:** Ina Coburger, Kefan Yang, Alisa Bernert, Eric Wiesel, Nirakar Sahoo, Sandip M. Swain, Toshinori Hoshi, Roland Schönherr, Stefan H. Heinemann

**Affiliations:** 1 Department of Biophysics, Center for Molecular Biomedicine, Friedrich Schiller University Jena and Jena University Hospital, Hans-Knöll-Str. 2, D-07745 Jena, Germany; 2 Department of Biology, The University of Texas Rio Grande Valley, 1201 West University Drive, Edinburg, TX 78539 USA; 3 Present Address: Department of Medicine, Duke University and Durham VA Medical Centers, Durham, NC 27710 USA; 4 Department of Physiology, University of Pennsylvania, 415 Curie Boulevard, Philadelphia, PA 19104-6085 USA

**Keywords:** K^+^ channel inactivation, A-type channel, β subunit, Hemin, Heme, Patch clamp

## Abstract

N-type inactivation of voltage-gated K^+^ channels is conferred by the N-terminal “ball” domains of select pore-forming α subunits or of auxiliary β subunits, and influences electrical cellular excitability. Here, we show that hemin impairs inactivation of K^+^ channels formed by Kv3.4 α subunits as well as that induced by the subunits Kvβ1.1, Kvβ1.2, and Kvβ3.1 when coexpressed with α subunits of the Kv1 subfamily. In Kvβ1.1, hemin interacts with cysteine and histidine residues in the N terminus (C7 and H10) with high affinity (EC_50_ 100 nM). Similarly, rapid inactivation of Kv4.2 channels induced by the dipeptidyl peptidase-like protein DPP6a is also sensitive to hemin, and the DPP6a mutation C13S eliminates this dependence. The results suggest a common mechanism for a dynamic regulation of Kv channel inactivation by heme/hemin in N-terminal ball domains of Kv α and auxiliary β subunits. Free intracellular heme therefore has the potential to regulate cellular excitability via modulation of Kv channel inactivation.

**Electronic supplementary material:**

The online version of this article (10.1007/s00424-020-02386-1) contains supplementary material, which is available to authorized users.

## Introduction

All voltage-gated potassium (Kv) channels share a similar overall structure with four α subunits, each consisting of six transmembrane helices and intracellular N and C termini. Kv channels open similarly with membrane depolarization but markedly differ with respect to their inactivation properties. N-type or fast inactivation is found in A-type potassium channels and is due to a “ball-and-chain” mechanism involving the N terminus. Thereby, the N terminus acts as a “ball” structure and occludes the ion permeation pathway [11]. In excitable cells, K^+^ channel inactivation regulates the shape of action potentials and the frequency of repetitive firing [5, 11]. N-type inactivation is subject to modulation by various cell signaling processes. For example, reactive oxygen species [32], pH [26], hydrogen sulfide (H_2_S) [39], Ca^2+^-dependent phosphorylation [31], and heme/hemin [34] regulate N-type inactivation of Kv1.4 channels, which are expressed in various cell types, such as smooth muscles, neurons, and cardiac myocytes [3, 7, 35]. Upregulation during cardiac hypertrophy and heart failure mark Kv1.4’s clinical importance [15, 17, 22]. Kv3.4, another member of A-type channels, is present abundantly in skeletal muscles but also in mammalian neurons where it modulates action potential duration and repolarization rate [29]. “Delayed-rectifier” Kv channel subunits, including Kv1.1 and Kv1.5, lack an N-terminal inactivation ball domain and by themselves undergo little or very slow inactivation [12, 19, 24]. However, such Kv1-containing channels do undergo fast N-type inactivation when complexed with auxiliary cytoplasmic β subunits (Kvβ) [10, 28]. Their variable N-terminal ball domains attached to the highly conserved core domain confer fast N(β)-type inactivation once bound to Kv1 α subunits [28], thus greatly increasing the diversity of Kv channel inactivation phenotypes.

A phenomenon similar to N(β)-type inactivation is observed in Kv4.2 channels: transmembrane dipeptidyl peptidase-like proteins, such as DPP6 and DPP10, function as β subunits for Kv4 channels. Although their major protein part is located on the extracellular side, their N termini face the cytosol where they also induce fast channel inactivation [1, 21, 37].

Heme, Fe(II) protoporphyrin IX, is an essential cofactor in many hemoproteins and plays important roles in catalytic processes as well as in binding and transport of gases such as O_2_ [6, 25]. In addition to this covalently bound form, free heme also interacts with proteins in a more dynamic manner by binding to heme-regulatory motifs (HRM) and thus functions as an intracellular signaling molecule [16]. Thereby, free or “labile” heme modulates the activity of diverse signal transducers and transcriptional regulators, such as the iron regulator regulatory protein (Irr), the heme activator protein (Hap1), and components of the Ras-MAPK signaling pathway [21]. By direct interaction with ion channels, heme may also have an impact on the cellular electrical excitability. For example, depending on the membrane voltage, heme and also the ferric hemin (Fe^3+^-containing protoporphyrin IX) activate or inhibit the large-conductance voltage- and Ca^2+^-dependent potassium channel (Slo1 BK). A cytochrome-C-like CKACH motif in the C terminus constitutes one heme binding site [37]. In addition, heme impairs the fast N-type inactivation of Kv1.4 channels by binding to cysteine and histidine residues in the N-terminal ball domain [34].

The presence of cysteine and histidine residues in the inactivation domains of other Kv α subunits (Kv3.4) and some auxiliary β subunits (Kvβ1.1, Kvβ1.2, Kvβ3.1) (Suppl. Fig. 1) suggests that heme might be a more general modulator of N-type and N(β)-type inactivation. Here, we describe the impact of hemin on the inactivation induced by the N termini of Kv3.4 α subunits and the auxiliary β subunits Kvβ and DPP6a, acting on Kv1 and Kv4 channels, respectively. Site-directed mutagenesis together with electrophysiology and microscale thermophoresis (MST) of recombinantly produced proteins demonstrate that N-terminal cysteine and histidine residues take part in hemin coordination in the nanomolar concentration range. The results suggest a general mechanism of select A-type Kv channel modulation by heme or hemin with a potential influence on cellular excitability.

## Materials and methods

### Chemicals

Solutions were made of high-grade chemicals obtained from Sigma-Aldrich (Taufkirchen, Germany) and Carl Roth (Karlsruhe, Germany). Stock solutions (1 mM) of hemin (Fe(III) protoporphyrin IX) and protoporphyrin IX (ppIX) were prepared daily by dissolving them in 30 mM NaOH for 30 min and were stored at 4 °C in the dark. Working solutions were diluted from 1 mM stock in bath solution immediately before use.

### Channel constructs and mutagenesis

The expression plasmids coding for Kv1.4 (Kcna4, P15385) and Kv3.4 from *Rattus norvegicus* (Kcnc4, Q63734) and mutants were cloned as described before [34, 39]. The expression plasmids encoding Kv1.5 (KCNA5, P22460), Kvβ1.1, Kvβ1.2, Kvβ1.3 (KCNAB1, Q14722), Kvβ3.1 (KCNAB3, O43448), and DPP6a from *Homo sapiens* and Kv1.1 (Kcna1, P10499) and Kv4.2 (Kcnd2, Q63881) from *Rattus norvegicus* were subcloned into pcDNA3.1. Accession numbers refer to the UniProt database. Mutations were generated using the QuikChange Site-Directed Mutagenesis Kit (Agilent, Waldbronn, Germany) or an overlap-extension mutagenesis approach [33].

For protein expression in *Escherichia coli*, the sequence coding for hKvβ1.1 amino acids M1-K140 was subcloned into a modified pMALc2T vector with an N-terminal maltose-binding protein (MBP) followed by a (His)_6_-tag. Mutations were created using the QuikChange Site-Directed Mutagenesis Kit (Agilent) and verified by sequencing.

### Channel expression in HEK293t cells

Human embryonic kidney 293t (HEK293t) cells (CAMR, Porton Down, Salisbury, UK) were cultured in medium composed of 45% Dulbecco’s minimal Eagle’s medium (DMEM) and 45% Ham’s F-12 Nutrient Mixture, supplemented with 10% fetal bovine serum in a humid 37 °C incubator with 5% CO_2_. When HEK293t cells were grown to 30–50% confluence, they were transiently transfected with plasmid coding for Kv α subunits alone or with β subunits using the Roti-Fect transfection kit (Carl Roth, Karlsruhe, Germany). The weight ratio between α and β subunits DNA for transfection was 1:3. For visual identification of transfected cells, CD8 plasmid was cotransfected, and anti-CD8-coated Dynabeads (Deutsche Dynal GmbH, Hamburg, Germany) were used. Electrophysiological recordings were performed 1–2 days after transfection.

### Channel expression in *Xenopus* oocytes

Expression in *Xenopus laevis* oocytes and recording from inside-out patches is described in the Supplementary Material.

### Electrophysiological recordings

Inside-out recordings were performed at room temperature (20–24 °C) using an EPC-9 or EPC-10 patch-clamp amplifier operated by PatchMaster software (both HEKA Elektronik, Lambrecht, Germany). Patch pipettes were fabricated from borosilicate glass (GB150F-8P, Science Products, Hofheim, Germany) and were coated with dental wax (Patterson Dental, Mendota Heights, MN, USA) to reduce their capacitance. After fire-polishing the pipettes, resistances of 0.9–2.5 MΩ were obtained. An agar bridge connected the bath solution and the ground electrode. All voltages were corrected for the liquid junction potential. Leak and capacitive currents were corrected using a p/6 method. Depending on the kinetics of recovery from inactivation, test pulses of Kv1.4 and Kv1.4/Kvβ1 were typically applied every 60 s, and every 15 s for Kv3.4 and Kv4.2.

The pipette solution composed of (in mM) 148 *N*-methyl-d-glucamine (NMDG), 10 KCl, 1 MgCl_2_, 1.5 CaCl_2_, and 10 4-(2-hydroxyethyl)-1-piperazineethanesulfonic acid (HEPES), pH 7.9 with HCl. Ten millimolar K^+^ was used to minimize the impact of C-type inactivation [19, 27]. The bath solution composed of (in mM) 140 KCl, 0.2 reduced glutathione (GSH), 10 ethylene glycol tetraacetic acid (EGTA), and 10 HEPES, pH 7.9 with KOH. For Kv4.2 recordings, the pipette solution contained (in mM) 146 NaCl, 4 KCl, 2 MgCl_2_, 2 CaCl_2_, 0.2 GSH, and 10 HEPES, pH 7.4 with NaOH.

### Expression and purification of hKvβ1.1

hKvβ1.1 comprising amino acids M1-K140 was expressed in *Escherichia coli* BL21 (*DE3*) pRIL. Cells were grown in TY medium at 22 °C to mid-log growth phase, induced with 0.5 mM IPTG (isopropyl β-D-1-thiogalactopyranoside), and harvested after 20 h by centrifugation at 4000 *g* for 20 min. Cells were resuspended in buffer A (50 mM Tris, 500 mM NaCl, 5 mM DTT, pH 8.0) and lysed by sonification. The clear cell lysate was applied to a HisTrap FF crude affinity column (GE Healthcare), washed with buffer A + 16 mM imidazole, and eluted with buffer A + 250 mM imidazole. The protein was further purified using a SD200 10/300 Increase column (GE Healthcare) equilibrated with PBS, pH 7.4, 1 mM TCEP (tetrachlorphenole).

### Microscale thermophoresis

Microscale thermophoresis (MST) experiments were performed with the Monolith NT.115 (NanoTemper Technologies) in a buffer containing PBS, 2 mM GSH, pH 7.4. Purified hKvβ1.1 1–140 was labeled according to the manufacturer’s instructions using the Labelling Kit Monolith NT RED-NHS (NanoTemper Technologies). A twofold dilution series of hemin ranging from 1.2 nM to 40 μM was mixed with labeled protein (final protein concentration: approximately 50 nM). To remove aggregates, each sample was centrifuged at 13,000 rpm for 5 min before filled into premium capillaries (NanoTemper Technologies).

### Data analysis and statistics

Data were analyzed with FitMaster (HEKA Elektronik) and IgorPro (WaveMetrics, Lake Oswego, OR, USA). Data are presented as means ± SEM with *n* independent measurements.

## Results

### Impact of hemin on Kv3.4 inactivation

**Fig. 1:**
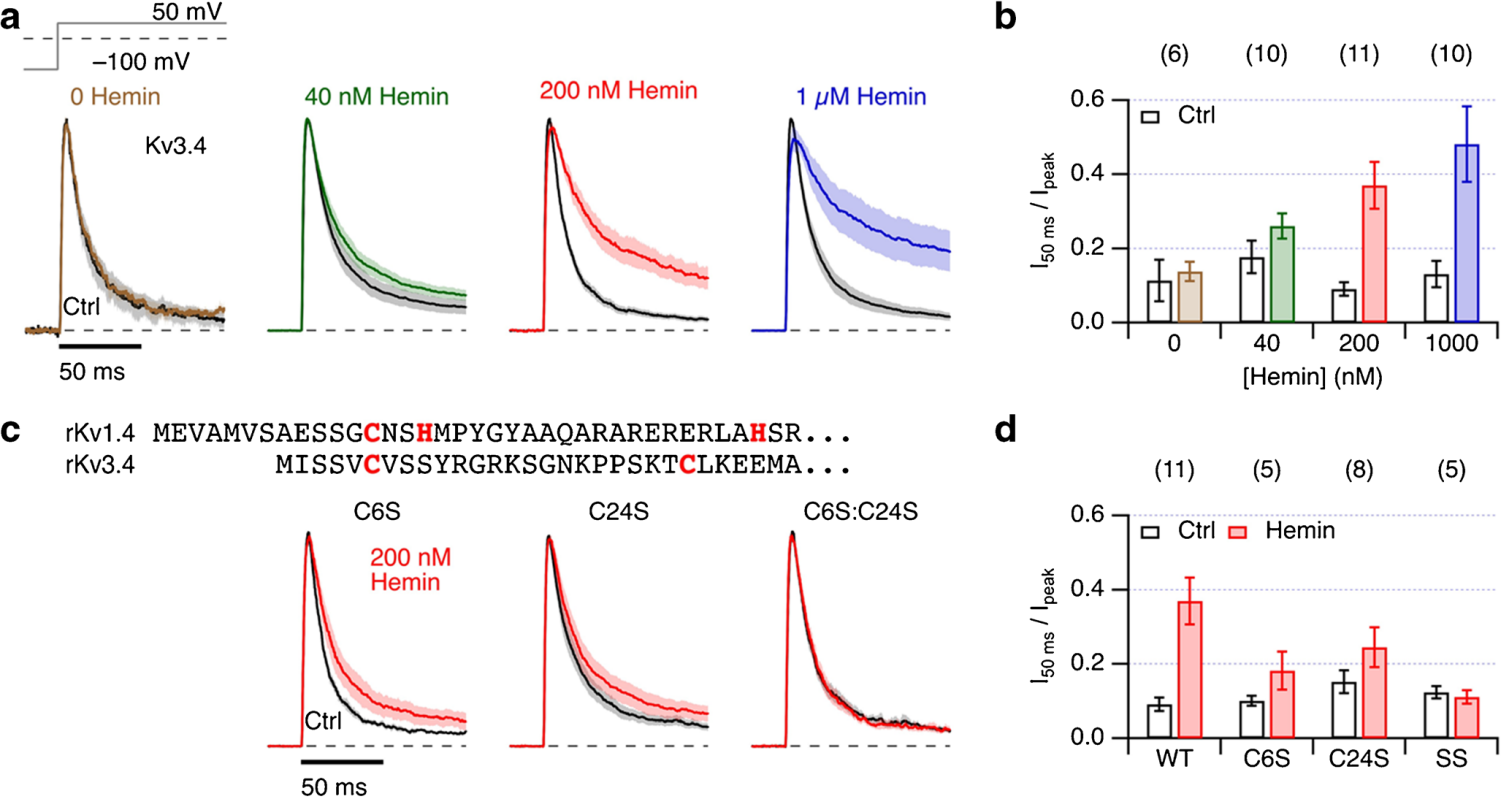
Inactivation of Kv3.4 is impeded by hemin. **a** Mean inside-out patch-clamp current traces, normalized to the peak current, from HEK293t cells expressing Kv3.4 channels for depolarization steps to 50 mV from a holding potential of − 100 mV in solutions containing 200 μM reduced GSH about 1 min after patch excision (Ctrl, black) and about 2 min after application of the same control solution (*left*) or solutions containing the indicated hemin concentration (color). Thick traces are mean values and shading indicates SEM. For *n,* see panel (**b**). **b** Mean non-inactivated current fraction at 50 ms after depolarization onset under control conditions (white bars) and for the indicated hemin concentrations (color). Data are means ± SEM with *n* in parentheses. **c** Alignment of the N-terminal protein sequences of (rat) rKv1.4 and rKv3.4 α subunits with Cys and His highlighted (*top*). Mean normalized current traces as in (**a**) for rKv3.4 mutants C6S, C24S and the combination C6S:C24S(SS) for application of 200 nM hemin (*bottom*). **d** Mean non-inactivated current fraction for 200 nM hemin application to the indicated mutants

Kv1.4 channels undergo both N- and C-type inactivation. The fast N-type inactivation is markedly slowed down by intracellular hemin, and molecular analysis revealed hemin binding to the N-terminal ball domain via interaction with cysteine and, to a lesser extent, histidine residues [34]. Since Kv3.4 channels undergo even faster N-type inactivation, which is also redox sensitive [39], we expressed Kv3.4 in HEK293t cells, measured voltage-activated currents in inside-out membrane patches, and applied hemin. As shown in Fig. 1a, Kv3.4 produced voltage-dependent K^+^ currents with rapid inactivation properties with an inactivation time constant at 50 mV of about 13 ms, albeit variable among different cell batches. To avoid a confounding effect by oxidizing cysteines in the N-terminal inactivation domain, the intracellular solution contained 200 μM reduced GSH. Under this condition, application of 200 nM hemin in the intracellular solution considerably slowed down the inactivation of Kv3.4 channels with time constant of inactivation increasing from 12.0 ± 0.1 ms (GSH control) to 23.8 ± 0.5 ms (200 nM hemin); the non-inactivating current component increased from 5.2 ± 0.1 to 22.0 ± 0.2%. At 1 μM hemin, inactivation was impaired even more (28.6 ± 0.2 ms and a non-inactivating component of 34.9 ± 0.1%), and even at 40 nM hemin, there was a consistent slowing effect (Fig. 1a, b). The hemin effect at 200 nM saturated after about 1.5 min. Washout of the hemin for 2 min resulted in partial recovery; additional DTT application resulted in more complete recovery (Suppl. Fig. 2). Other than the aforementioned slow-down of inactivation, intracellular hemin had no impact on the recovery from inactivation (Suppl. Fig. 3) and the peak current-voltage relationship (Suppl. Fig. 4). The peak current was only marginally affected (Suppl. Fig. 5). Protoporphyrin IX (ppIX), i.e., the ring structure without a central metal ion, even at 2 μM neither slowed down inactivation nor inhibited the effect of 200 nM hemin on Kv3.4 channel inactivation (Suppl. Fig. 6).

The N terminus of Kv3.4 harbors two cysteine residues (C6 and C24), which were previously shown to contribute to the channel’s redox and hydrogen sulfide sensitivity [1, 32]. However, in contrast to Kv1.4, the Kv3.4 N terminus lacks histidine (Suppl. Fig. 1, Fig. 1c). Since cysteine residues often take part in heme coordination, we mutated them individually and together to serine (C6S, C24S, C6S:C24S) and examined the effect of 200 nM hemin. For the single mutants, the impact of 200 nM hemin was strongly diminished (Fig. 1c, d): the non-inactivated current fraction after 50 ms at 50 mV decreased from 0.35 (WT) to less than 0.25 for both single mutants. The double mutant C6S:C24S was insensitive to hemin. In neither case, the peak currents were significantly affected (Suppl. Figs. 4, 5). These results suggest that both cysteine residues in the N-terminal ball domain contribute to hemin coordination, which subsequently impairs the N-type inactivation process of Kv3.4 channels.

### Effect of hemin on Kvß1.1-induced inactivation

N termini of some A-type potassium channels contain heme binding motifs. In addition, auxiliary subunits of the Kvβ protein family harbor cysteines and histidines in their N termini (Suppl. Fig. 1), which potentially contribute to heme binding. The contribution of the Kvβ1 subunit is most readily assessed by coexpression with a non-inactivating Kv1 channel, such as Kv1.1. Expression of Kv1.1 in HEK293t cells, however, was not high enough to warrant inside-out macroscopic current patch-clamp measurements. Therefore, we coexpressed Kv1.1 together with Kvβ1.1 in *Xenopus* oocytes and measured K^+^ currents in inside-out macro patches. Expression of Kv1.1 alone without Kvβ1.1 resulted in non-inactivation currents after depolarization to 40 mV, and intracellular application of 200 nM hemin did not affect the current signal (Suppl. Fig. 7, *top*). When coexpressed with Kvβ1.1, the channel inactivated rapidly, and this inactivation was abolished after the application of 200 nM hemin (Suppl. Fig. 7). Kvβ1.1 harbors a _7_CxxH_10_ heme recognition site in the N-terminal ball domain similar to Kv1.4 (Suppl. Fig. 1). Mutagenesis of C7S and H10A in isolation resulted in a clearly diminished effect of hemin on inactivation, and only concurrent double mutation (C7S:H10A) rendered the Kvβ1.1-induced inactivation of Kv1.1 channels insensitive to 200 nM hemin (Suppl. Fig. 7, *bottom*).

**Fig. 2:**
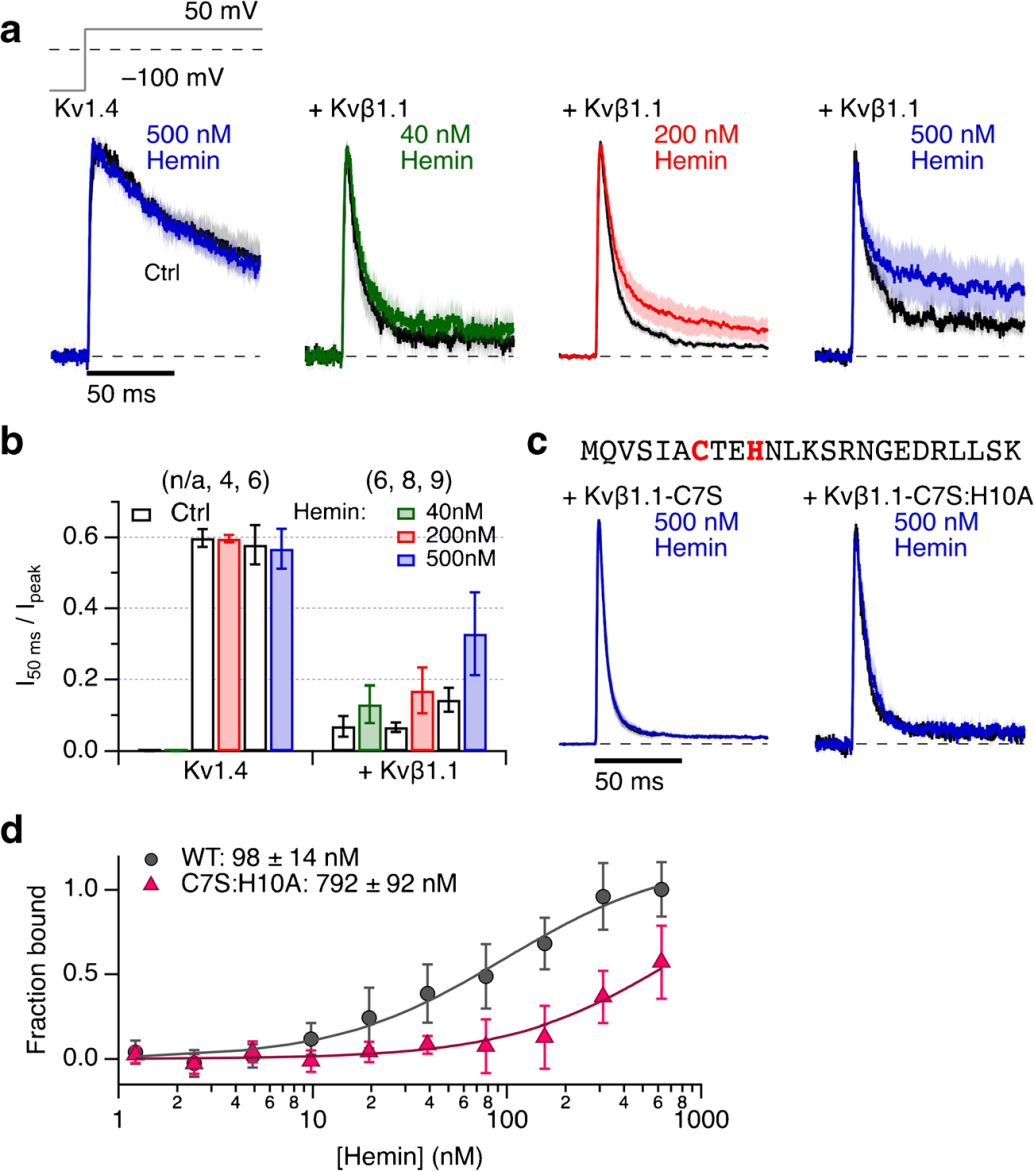
Heme sensitivity of Kvβ1.1-mediated inactivation. **a** Mean normalized inside-out patch-clamp current traces from HEK293t cells expressing Kv1.4 channels alone (*left*) or with coexpression of Kvβ1.1 by depolarization steps to 50 mV from a holding potential of − 100 mV. Thick black traces are means before, and the colored traces about 2 min after application of the indicated concentrations of hemin. All solutions additionally contained 200 μM GSH. Shading indicates SEM. For *n*, see panel (**b**). **b** Fraction of non-inactivated current after 50 ms depolarization for Kv1.4 and with coexpression of Kvβ1.1. Data are means ± SEM, *n* in parentheses. **c** N-terminal protein sequence of Kvβ1.1 (*top*). Current traces as in (**a**) for Kvβ1.1 mutants C7S and C7S:H10A (*bottom*). Measurements were performed at pH 7.9 to eliminate N-type inactivation endogenous to Kv1.4. **d** Microscale thermophoresis of the Kvβ1.1 N-terminal domain. Binding curves for interaction of Kvβ1.1 1–140 fused to MBP (gray circles) and mutant C7S:H10A (magenta triangles) as a function of hemin concentration, normalized to the WT data at the highest concentration of hemin. Data are means ± SEM (*n* = 3; 2 protein preparations)

To better compare with the results obtained for Kv3.4, we also studied heme–Kvβ interaction in HEK293t cells. To yield large enough currents, we expressed Kv1.4 channels alone and in combination with Kvβ1.1 subunits and mutants (Fig. 2). As we showed previously [34], N-type inactivation of Kv1.4 is sensitive to intracellular hemin. However, at high pH, this N-type inactivation kinetics is too slow to be discerned from residual C-type inactivation [26, 39]. Therefore, when measuring at pH 7.9, the Kv1.4 currents recorded in inside-out patches exhibit slow inactivation that is insensitive to intracellular hemin application (Fig. 2a). In addition, basic pH increases the solubility of hemin. When coexpressed with Kvβ1.1, however, the inactivation time course was much accelerated and became sensitive to hemin. Hemin at 200 nM clearly slowed down inactivation at 50 mV, and even at 40 nM, some slowing was observed (Fig. 2a, b). The single mutation C7S and the double mutation C7S:H10A in Kvβ1.1 rendered the Kv1.4 complexes insensitive to 500 nM hemin (Fig. 2c). Only for hemin-sensitive channel complexes intracellular hemin application slightly increased the peak current as expected for partial removal of inactivation (Suppl. Fig. 8).

### Hemin binds with high affinity to Kvß1.1

To confirm the physical interaction of hemin with the N terminus of Kvβ1.1 suggested by the electrophysiological results, microscale thermophoresis was used to evaluate the binding strength of hemin to the recombinant Kvβ1.1 1–140 protein fused to MBP-(His)_6_. Titration with hemin against labeled hKvβ1.1 1–140 fused to MBP-(His)_6_ induced clear temperature-dependent changes in fluorescence (Fig. 2d). Determination of the binding affinity revealed a binding constant of 98 ± 14 nM (Fig. 2d). Performing the same analysis for protein mutant C7S:H10A showed no binding signal up to about 100 nM hemin. Since the maximally accessible hemin concentration is limited by progressive fluorescence quenching, experiments with MST are restricted to about 600 nM hemin, thus precluding the determination of saturation for the mutant. With a data fit constrained to the maximally obtainable MST signal, we thus estimated a hemin binding constant to the mutant to be 792 ± 92 nM (Fig. 2d). It should be noted that this is a lower limit and may also reflect unspecific binding to the protein.

### Splice variants of Kvß1 subunits

**Fig. 3:**
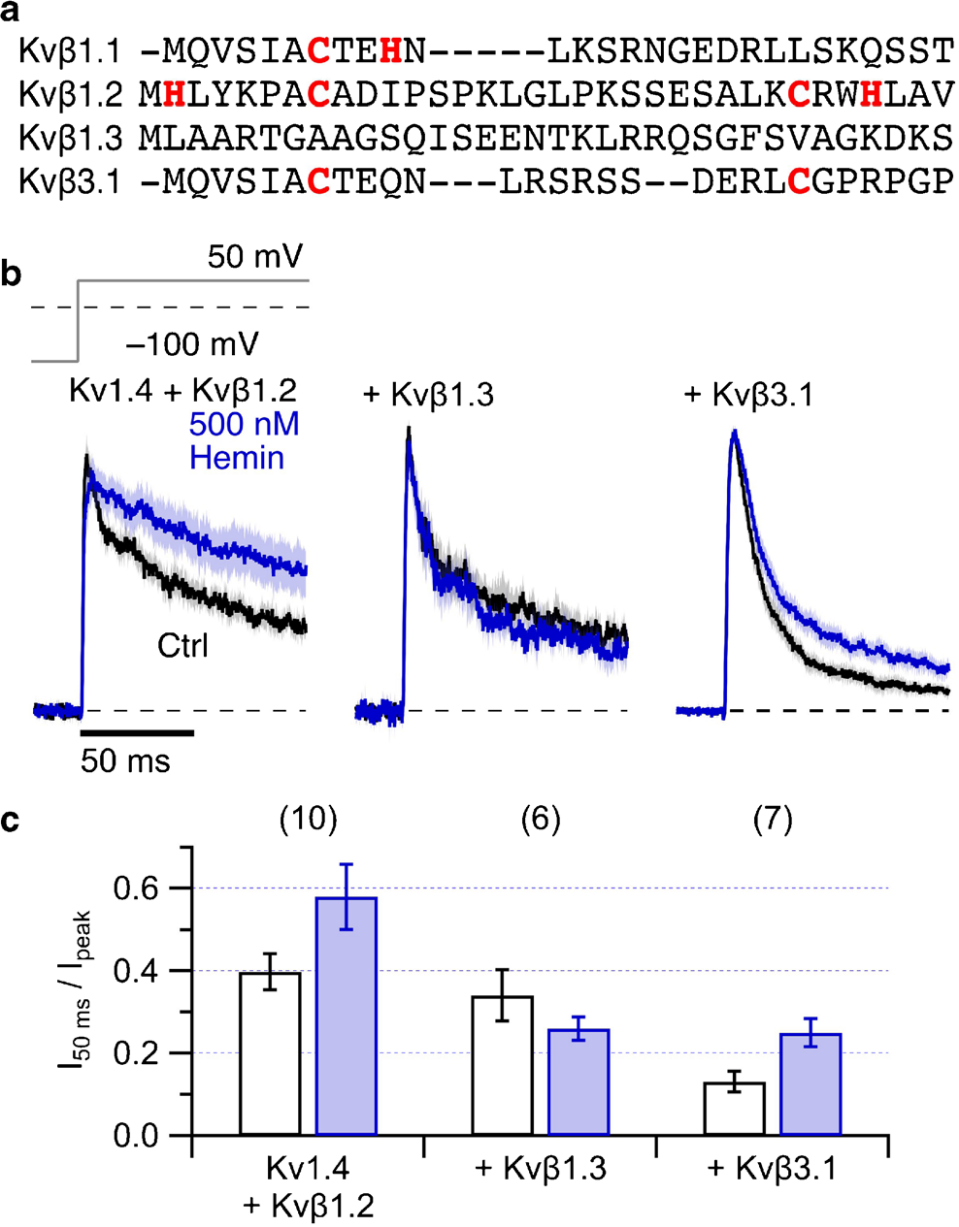
Kv1.4 coexpression with Kvβ1.2, Kvβ1.3, and Kvβ3.1. **a** N-terminal protein sequence alignment of the Kvβ1 splice variants and Kvβ3.1. **b** Mean normalized inside-out patch-clamp current traces from HEK293t cells expressing Kv1.4 channels with Kvβ1.2 (*left*), Kvβ1.3 (*center*), or Kvβ3.1 (*right*) by depolarization steps to 50 mV from a holding potential of − 100 mV. Thick black traces are means before and the colored traces about 2 min after application of 500 nM hemin (blue). All solutions additionally contained 200 μM GSH. Shading indicates SEM. For *n*, see panel (**c**). Measurements were performed at pH 7.9 to eliminate N-type inactivation endogenous to Kv1.4. **c** Fraction of non-inactivated current after 50 ms depolarization for Kv1.4 with coexpression of the indicated Kvβ subunits. Data are means ± SEM, *n* in parentheses

The splice variants Kvβ1.1-Kvβ1.3 differ in their N-terminal inactivation domains. While Kvβ1.1 harbors the heme-regulatory motif _7_CxxH_10_ in the distal ball domain, Kvβ1.2 exhibits a similar motif (_28_CxxH_31_) in the “chain” domain. In addition, the N-terminal ball domain of Kvβ1.2 contains a histidine and a cysteine that are separated by five residues (_2_H…C_8_, Fig. 3a). The N terminus of Kvβ1.3 does not harbor cysteine or histidine (Fig. 3a). We thus examined heme dependence of inactivation induced by Kvβ1.2 and Kvβ1.3 when coexpressed with Kv1.4 in HEK293t cells. In this combination, however, Kvβ1.2 only resulted in a marginal acceleration of Kv1.4 inactivation. Application of 500 nM intracellular hemin slowed down inactivation further to a level of Kv1.4 channels alone (Fig. 3b, c, *left*). Coexpression of Kv1.4 with Kvβ1.3 results in somewhat faster inactivation, and its time course was unaffected by 500 nM hemin (Fig. 3b, c, *center*). Coexpression of Kv1.5 and Kvβ1.3 in *Xenopus* oocytes and application of 200 nM hemin to inside-out patches revealed that hemin neither affects Kv1.5 channels alone or N-type inactivation induced by Kvβ1.3 (Suppl. Fig. 9). Coexpression of Kvβ3.1, which harbors two cysteine residues in the N-terminal domain (Fig. 3a), with Kv1.4 also results in faster inactivation than Kv1.4 alone, and 500 nM hemin slowed down inactivation (Fig. 3b, c, *right*). In general, the impact of hemin on the inactivation of Kvβ-mediated inactivation appeared weaker than that on N-type inactivation of Kv3.4.

### Fast inactivation of Kv4.2 channels

**Fig. 4:**
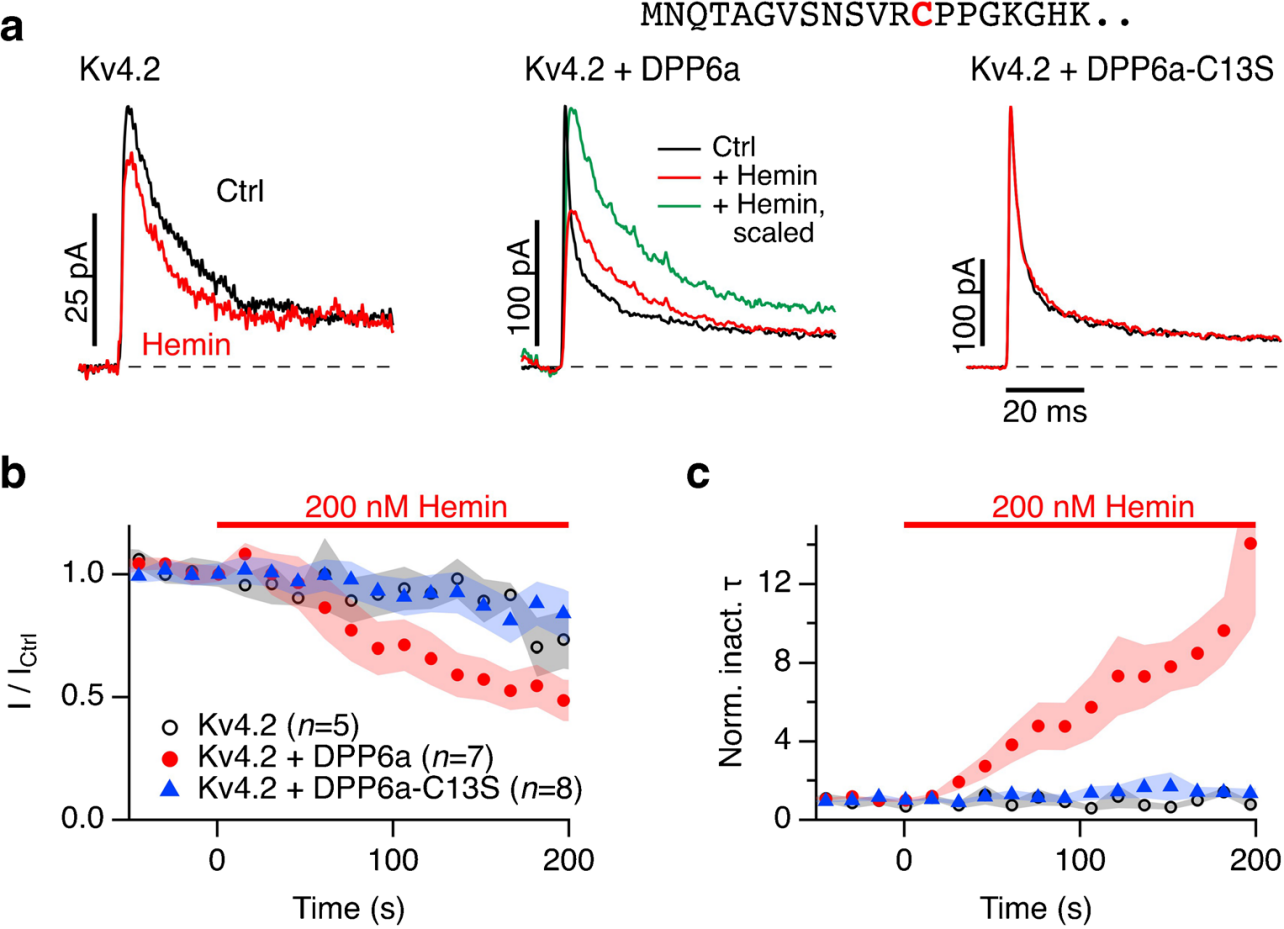
Impact of hemin on inactivation induced by DPP6a. **a** Representative inside-out current recordings from HEK293t cells expressing Kv4.2 alone (*left*) or together with human DPP6a (*center*) or its mutant C13S (*right*). Currents were measured at 60 mV before (black) and 200 s after application of 200 nM hemin in the presence of 200 μM reduced GSH (red). The green trace in the center panel is the trace with hemin but scaled in amplitude to match the peak current of the control. The N-terminal sequence of human DPP6a is shown at the top. **b** Time course of the peak currents for the indicated constructs and hemin application. **c** Time constants of fast inactivation, normalized to the control values before hemin application. Symbol use and *n* as in (**b**). Data in (**b**) and (**c**) are means ± SEM with *n* indicated in (**b**)

K^+^ channels of the Kv4 family also exhibit rapid inactivation, which is further accelerated by coassembly with transmembrane dipeptidyl peptidase-like proteins, such as DPP6 or DPP10. The N terminus of these single-pass transmembrane proteins reaches the cytosolic face to function as inactivation accelerator [13]. Since the N-terminal sequences of DPP6a and DPP10a contain cysteine and histidine residues that may potentially coordinate heme (e.g., Fig. 4a, *top*), we investigated Kv4.2 channels in HEK293t cells alone and coexpressed with DPP6a. When recorded in the whole-cell configuration, extracellular application of 5 μM hemin did not affect the current amplitude or the time course of inactivation (*n* = 5, data not shown). In the inside-out patch-clamp configuration, however, application of 200 nM hemin under reducing conditions eliminated the rapid inactivation induced by DPP6a to a level approximately corresponding to the inactivation seen with Kv4.2 alone (Fig. 4a, *left, center*). In addition, hemin application (200 nM) reduced the peak current amplitude to about 50% (Fig. 4b). Mutagenesis of DPP6a to eliminate the N-terminal cysteine (DPP6a-C13S) abolished both effects: Kv4.2 + DPP6a-C13S currents were insensitive to 200 nM hemin, both with respect to peak amplitude and time course of inactivation (Fig. 4).

## Discussion

N-type inactivation is a major characteristic of A-type potassium channels and can be modulated by several physiological factors [4, 6, 25, 32, 39]. In this study, we showed that fast inactivation of Kv3.4 is slowed by hemin in a probable physiologically relevant concentration range. Mutagenesis revealed that the cysteine residues C6 and C24 in the N-terminal ball domain are important for the heme dependence. Only mutagenesis of both cysteines to serine completely rendered fast N-type inactivation resistant to hemin (Fig. 1). Kv3.4 is widely expressed in the mammalian nervous system and regulates the repolarization of action potentials and thus its duration [18, 29, 30]. Slowing of inactivation by heme may, therefore, result in shorter and fewer action potentials, thus affecting electrical excitability of neurons. In a synapse, the changes in action potential duration may influence Ca^2+^-dependent neurotransmission. For example, in dorsal root ganglia (DRG) cells containing Kv1.4 and Kv3.4 channels, a physiological consequence would be an attenuation of pain signaling [8, 36].

The same reasoning may be applied to Kv1.4 channels in DRG cells. As we showed previously [34], heme also impairs N-type inactivation Kv1.4 channels. In this case, C13 and H16 take part in coordinating heme to affect the structure of the inactivating ball-and-chain domain [34]. Interestingly, the N-terminal inactivation domains of Kv3.4 and Kv1.4 exhibit no structural similarity. While the inactivation peptide of Kv3.4 exhibits a four-leaf clover-like structure [1], Kv1.4 harbors a flexible inactivation domain anchored at a five-turn helix [38]. Perhaps, specific sequence motifs are not required for heme–peptide interactions. Here, we identified cysteine residues in Kv3.4 to contribute to heme coordination (Fig. 1). It should be noted, though, that other residues, for example Tyr10, may be involved in stabilizing a heme–peptide complex.

Cysteine residues were identified to be important in the impact of hemin on Kv3.4 inactivation. The effect of hemin may also be mediated by coordination of heme/hemin by the inactivating peptide or potentially by redox processes involving C6 and C24. We tried to limit a potential redox process by performing the experiments in the presence of a 1000-fold excess of reduced glutathione; however, we cannot entirely exclude the possibility that the named cysteine residues undergo some chemical modification in the presence of ferric hemin.

Based on the results with Kv1.4 and Kv3.4, heme might also be a regulator of N(β)-type inactivation. Indeed, while elevated intracellular hemin was without effect on delayed rectifier Kv channels formed by Kv1.1 α subunits, inactivation induced by coexpression of Kvβ1.1 was readily impaired by hemin (Suppl. Fig. 7). The same was true for the Kvβ1.1-induced fast inactivation of Kv1.4 channels (Fig. 2). In the latter case, we took advantage of the pH dependence of the N-type inactivation endogenous to Kv1.4: at pH 7.9, Kv1.4 N-type inactivation is so slow such that it cannot be discriminated from C-type inactivation anymore, while inactivation due to Kvβ1.1 persists. Elimination of the potential heme-regulatory motif _7_CxxH_10_ in the ball domain of Kvβ1.1 rendered the inactivation resistant to hemin. Individual mutation of the cysteine and histidine in this motif furthermore revealed that both sites contribute, and only full elimination of the motif abolished the hemin effect completely. This situation is similar to what was found for Kv1.4 channels with its N-terminal HRM _13_CxxH_16_ [34]. Investigating the mobility of N-terminal protein fragments of Kvβ1.1 (residues 1–140) as a function of hemin concentration using an MST assay, we found evidence for the physical binding of hemin to this protein with an apparent binding constant of about 100 nM. No such binding was visible in peptides without an HRM (Fig. 2d). Besides inference about hemin binding to the peptide, the MST results also suggest that the heme–protein complex has a different thermophoretic mobility than the free components, arguing for heme-induced protein conformational changes. Using an array of other physical binding assays and molecular docking, we previously also concluded for the N terminus of Kv1.4 that heme may induce some structure to the intrinsically disordered protein fragment, which interferes with the required flexibility for inducing N-type inactivation [34].

Functional evaluation of the splice variants Kvβ1.2 and Kvβ1.3 in inside-out patches from mammalian cells is compromised by the fact that they do not speed up inactivation of Kv1.4 as much as Kvβ1.1. Nevertheless, the results obtained (Fig. 3, Suppl. Fig. 9) are consistent with the overall notion that cysteine and/or histidine residues take part in mediating the heme dependence of N(β)-type inactivation. Kvβ1.3 lacks N-terminal cysteine or histidine resides and its inactivation is not affected by intracellular hemin Kvβ1.2 harbors even two potential HRMs and, hence, hemin eliminates Kvβ1.2-induced inactivation. The same is true for Kvβ3.1 with two cysteine residues in the N-terminal domain. On the other hand, Kvβ2.1 which lacks the inactivation peptide and critical heme coordinating amino acids in the N terminus should be not affected by heme.

The rapid inactivation induced by the dipeptidyl peptidase-like protein DPP6a to Kv4.2 channels is also sensitive to intracellular heme, while the inactivation endogenous to Kv4.2 channels is insensitive (Fig. 4). Again, a cysteine residue in the N-terminal part of DPP6a (C13) is involved. This cysteine is part of a so-called CP motif and, together with a histidine five residues downstream, may form a novel heme-coordination site (_13_CPPGKGH_19_). The potentially heme-ligating cysteine and histidine enclose proline and glycine for maximal flexibility to even allow for hexacoordinated heme binding. Although the exact mechanism of how DPP6a induces inactivation of Kv4.2 channels is still elusive, the existence of HRMs in the N terminus of DPP6a and the dependence on intracellular heme suggests at least some similarity to Kvβ subunits.

Slow-down or elimination of fast K^+^ channel inactivation by elevated intracellular free heme may impair spike broadening regulation, potentially influencing neuronal excitability and learning [9, 14]. Furthermore, heme deficiency observed in aging and Alzheimer’s disease [2] may influence the inactivation phenotype in the opposite direction.

Inactivation characteristics of mammalian Kv channel complexes vary markedly. Some Kv α subunits contain N-terminal inactivation domains with HRMs conferring heme sensitivity to their fast inactivation. Other delayed rectifier-type α subunits lack their own inactivation domains but assembly with select Kvβ subunits with HRMs may confer heme-sensitive N-type inactivation. Possibly, directed expression of Kvβ1.1, Kvβ1.2, or Kvβ3.1 on the one hand or Kvβ1.3 on the other generates inactivating K^+^ channels dependent or independent, respectively, of redox conditions and heme concentration.

The physiological or pathophysiological conditions under which cytosolic free heme concentrations are altered strongly enough to have an influence on K^+^ channel function, however, remain to be elucidated. The heme dependence of Kv3.4 and Kv1.4 + Kvβ1.1 and Kv1.1 + Kvβ1.1 complexes below 100 nM suggests that the binding properties are in a range that are discussed to be relevant to the modulation of the heme-dependent transcription repressor Bach1 [23]. Episodes of high heme concentrations such as in a trauma situation or after hemorrhagic insults may also affect electrical signaling by modulating the function of A-type channels. However, the exact physiological and/or pathophysiological significance is yet to be established, which requires quantitative assessments of free heme concentrations in live cells under different conditions. It is conceivable to utilize the heme binding sites at N-type inactivating channels as pharmacological targets to deliberately affect the speed of K^+^ channel inactivation, such as demonstrated for low-molecular weight K^+^ channel disinactivators [20].

Our results show that N-type inactivation mediated by cysteine/histidine containing N-terminal protein parts of A-type potassium channel α and auxiliary β subunits is impeded by hemin. The absence of sequence homology in the N-terminal binding regions suggests a general mechanism for heme-mediated modulation of A-type channel inactivation and, hence, of heme influencing electrical excitability.

## Electronic supplementary material

ESM 1 (PDF 1377 kb)

## Abbreviations

DTT: Dithiothreitol
DPP: Dipeptidyl peptidase-like protein
DRG: Dorsal root ganglia
GSH: Glutathione
HRM: Heme-regulatory motif
Kv channel: Voltage-gated potassium channel
MBP: Maltose-binding protein
MST: Microscale thermophoresis
ppIX: Protoporphyrin IX
WT: Wild type
