# Supplementary material for: Impact of intracellular hemin on N-type inactivation of voltage-gated K+ channels
Source: Pflugers Arch. 2020 May 10;472(5):551–60. doi: 10.1007/s00424-020-02386-1 (PMC7239824; doi:10.1007/s00424-020-02386-1)
Supplement: Supplementary file 1 — (PDF 1377 kb) [file 424_2020_2386_MOESM1_ESM.pdf]

# Impact of intracellular hemin on N-type inactivation of voltage-gated K⁺ channels

Ina Coburger[1#], Kefan Yang[1#], Alisa Bernert[1], Eric Wiesel[1], Nirakar Sahoo[1,2], Sandip M. Swain[1a], Toshinori Hoshi[3], Roland Schönherr[1], Stefan H. Heinemann[1*]

[1] Center for Molecular Biomedicine, Department of Biophysics, Friedrich Schiller University Jena and Jena University Hospital, Hans-Knöll-Str. 2, 07745 Jena, Germany

[2] Department of Biology, The University of Texas Rio Grande Valley, 1201 West University Drive, Edinburg, TX 78539, USA

[3] Department of Physiology, University of Pennsylvania, 415 Curie Boulevard, Philadelphia, PA 19104-6085, USA

[#] shared first authorship

[a] present address: Department of Medicine, Duke University and Durham VA Medical Centers, Durham, NC, 27710, USA.

* Correspondence: Stefan.H.Heinemann@uni-jena.de

## Supplementary Material

## Supplementary Methods

### Channel constructs mRNA synthesis

The expression plasmids coding for human Kv1.1 (KNCA1, Q09470), Kv1.5 (KCNA5, P22460), Kvβ1.1, and Kvβ1.3 (KCNAB1, Q14722) were subcloned into pGEM-HE, and capped mRNAs were synthesized *in vitro* as described, using the mMessage mMachine kit (Ambion, Austin, TX, USA) [1]. Accession numbers refer to the UniProt database. For generation of Kvβ1.1 mutants, see main text.

### *Xenopus laevis* oocyte preparation and mRNA injection

Oocytes were surgically removed from the ovarian tissue of *Xenopus laevis* according to an institutionally approved protocol. The oocytes were defolliculated and microinjected with 50 nl of a solution containing mRNA. The weight ratios of mRNA coding for α and β subunits were about 1:2. Inside-out patch-clamp recordings were performed on upon removal of the vitelline membranes 2-4 days after mRNA injection.

### Current recording from *Xenopus laevis* membrane inside-out patches

Ionic currents were recorded in the inside-out configuration at room temperature using an EPC-9 patch-clamp amplifier operated with PatchMaster software (both HEKA Elektronik, Lambrecht, Germany). Currents were measured using aluminum silicate glass pipettes with resistances of about 1 MΩ, coated with dental wax to reduce the capacitance. The intracellular solutions contained (in mM) 100 K-aspartate, 15 KCl, 1 reduced glutathione (GSH), 10 ethylene glycol tetraacetic acid (EGTA), 10 4-(2-hydroxyethyl)-1-piperazineethanesulfonic acid (HEPES), pH 8.0 with KOH. The extracellular solution contained (in mM) 103.6 Na-aspartate, 11.4 KCl, 1.8 $CaCl_2$, 10 HEPES, pH 7.2 with NaOH. Hemin (Fe(III) protoporphyrin IX, Sigma-Aldrich), was prepared from stock solutions in KOH (30 mM) immediately before the experiments.

## References

1. Sahoo N, Goradia N, Ohlenschlager O, Schönherr R, Friedrich M, Plass W, Kappl R, Hoshi T, Heinemann SH (2013) Heme impairs the ball-and-chain inactivation of potassium channels. Proc Natl Acad Sci U S A 110:E4036-44.

## Supplementary Figures

```
Kv1.4  MEVAMVSAESSGCNSHMPYGYAAQARARERERLA--HSRR
Kv3.4  -------MISSVCVSSYRGRKSGNKPP---SKTCLKEEMA

Kvβ1.1 ------MQVSIACTEHN---LKSRNG---EDRLLSKQSST
Kvβ1.2 -----MHLYKPACADIPSPKLGLPKS-SESALKCRWHLAV
Kvβ1.3 -----MLAARTGAAGSQISEENTKLRRQSGFSVAGKDKSP

Kvβ3.1 -----MQVS-IACTEQN---LRSRSS---EDRLCGPRPGP
```

**Supplementary Figure 1. Amino acid alignment of inactivation "ball" domains of selected A-type $K^+$ channel α subunits and auxiliary β subunits.**
Putative heme-interacting amino acids (cysteine and histidine) are highlighted in red.

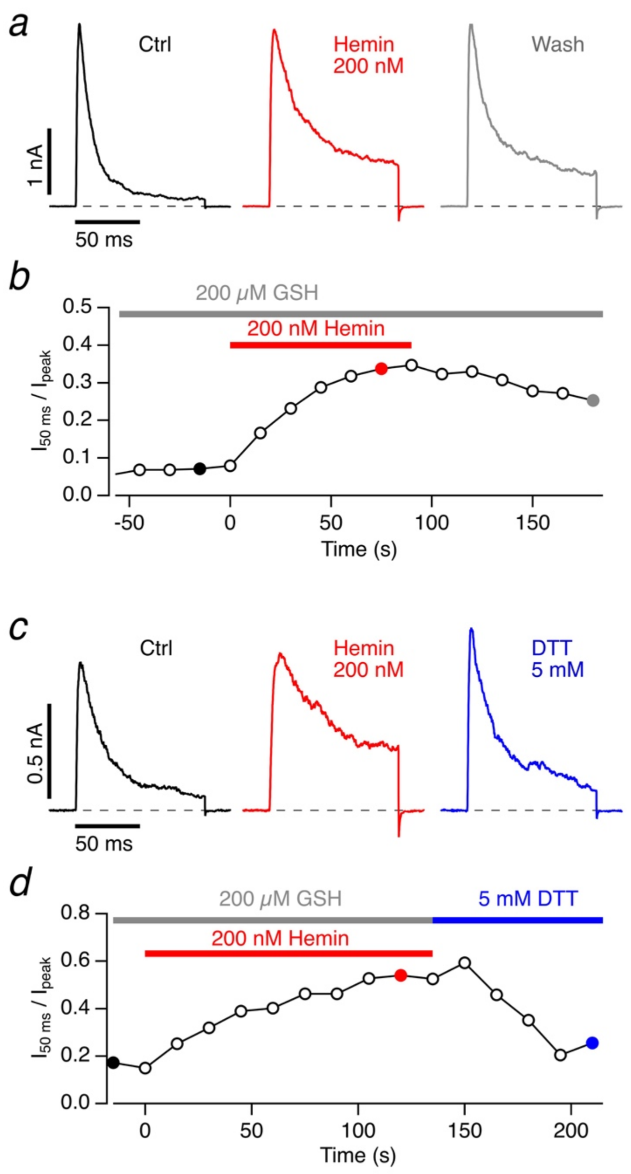


**Supplementary Figure 2. Onset and reversibility of hemin effect on Kv3.4 channels.** ***a*** Representative inside-out current traces at 50 mV from a holding potential of -100 mV for Kv3.4 channels in HEK293t cells in the presence of 200 μM GSH (Ctrl), after additional application of 200 nM hemin (red), and about 80 s after washout of the hemin (gray). ***b*** Time course of the inactivation index of the experiment shown in (*a*) with indication of 200 μM GSH and 200 nM hemin application. Straight lines connect data points for clarity. The marked symbols indicate the traces shown in (*a*). ***c***, ***d*** Similar experiments as in *a* and b with *a* washing buffer containing additional 5 mM DTT.

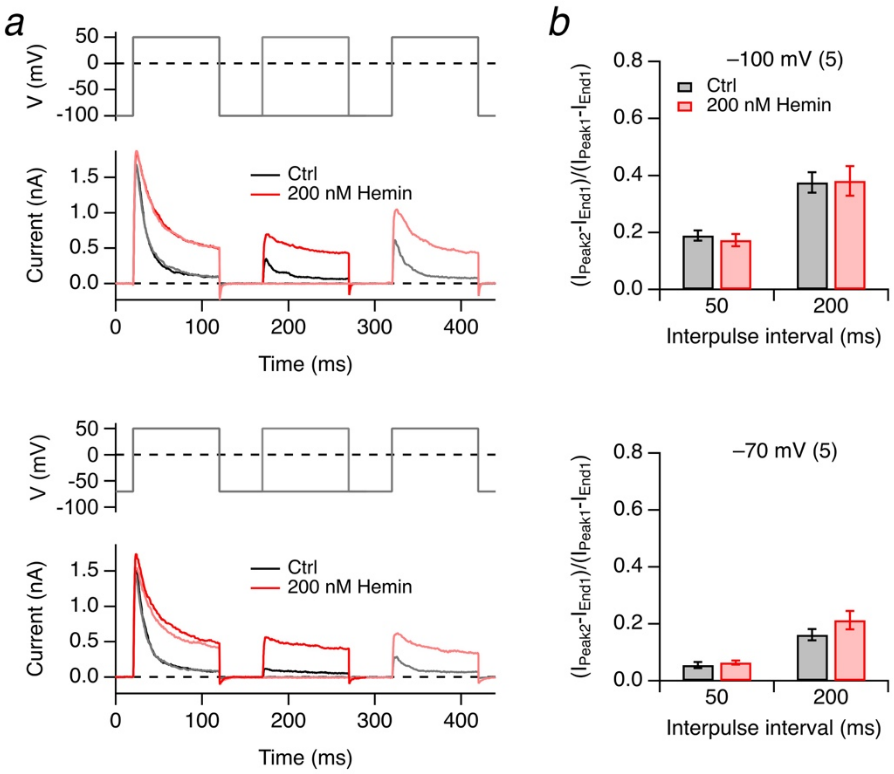


**Supplementary Figure 3. Kv3.4 channels: recovery from inactivation.**

***a*** Superposition of representative current recordings for the indicated pulse protocol from inside-out patches of HEK293t cells expressing Kv3.4 channels under control conditions (black) and in the presence of 200 nM hemin in 200 $\mu$M GSH (red traces) for holding potential and recovery episodes at -100 mV (*top*) and -70 mV (*bottom*).
***b*** Mean relative recovery from inactivation from data as shown in *a*. Data are means±SEM with *n* in parentheses.

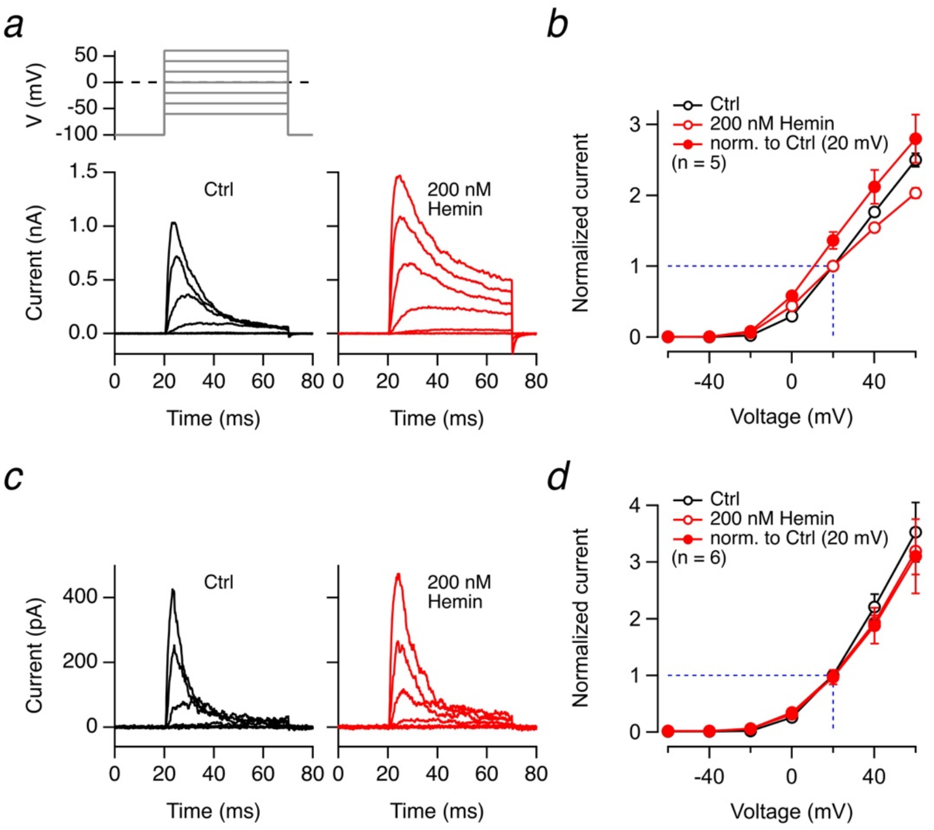


**Supplementary Figure 4. Kv3.4 channels: voltage dependence of activation.**
***a*** Superposition of representative current recordings for the indicated pulse protocol from inside-out patches of HEK293t cells expressing Kv3.4 channels under control conditions (black) and in the presence of 200 nM hemin in 200 $\mu$M GSH (red traces). ***b*** Mean peak current, normalized to the values at 20 mV, as function of voltage. The filled symbols indicate data in hemin normalized to the respective control data. Straight lines connect data points for clarity. ***c***, ***d*** As in *a* and *b* for Kv3.4-C6S:C24S. Data are means±SEM with *n* in parentheses.

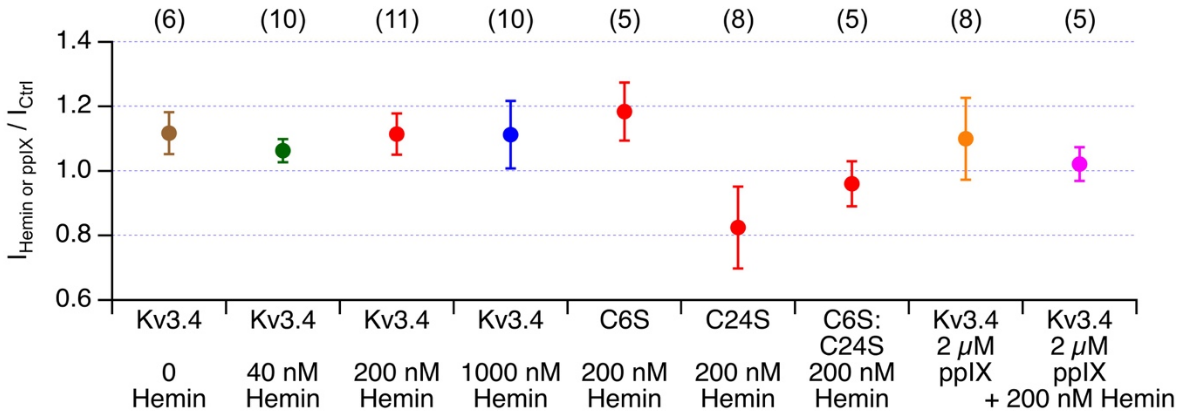


**Supplementary Figure 5. Fractional peak currents of Kv3.4 channels at 50 mV.**
Mean fractional change of the peak current of Kv3.4 channels and the indicated mutants in inside-out patches when subjected to hemin or protoporphyrin IX (ppIX) as indicated. Data are means±SEM with *n* in parentheses.

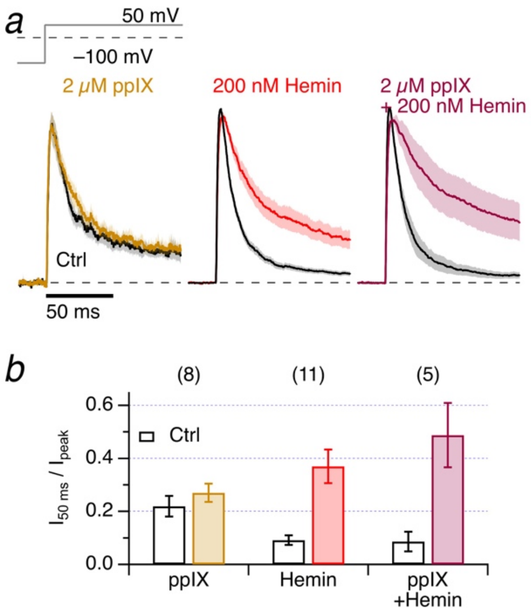


**Supplementary Figure 6. Protoporphyrin IX (ppIX) does not slow down inactivation of Kv3.4 channels.** ***a*** Mean normalized inside-out current traces at 50 mV from a holding potential of -100 mV for Kv3.4 channels in HEK293t cells in the presence of 200 $\mu$M GSH (black traces) and with additional application of 2 $\mu$M ppIX (brown), of 200 nM hemin (red), and both together (magenta). Thick traces are mean values and shading indicates SEM (for *n* see panel *b*). ***b*** Mean inactivation index at 50 ms of the data shown in (*a*) before (white bars) and after the 2 $\mu$M ppIX, 200 nM hemin, and both together. Data are means±SEM with *n* in parentheses.

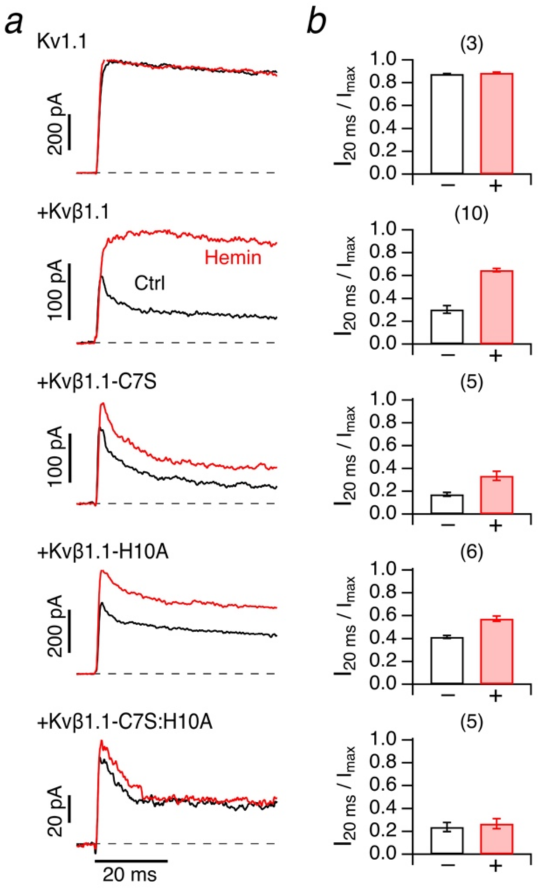


**Supplementary Figure 7. Kv1.1 and Kvβ subunits in *Xenopus* oocytes.**
***a*** Representative current traces at 40 mV from inside-out patches of *Xenopus* oocytes expressing Kv1.1 channels alone (*top*) or Kv1.1 and the indicated Kvβ subunits before (black) and about 150 s after application of 200 nM hemin to the intracellular side of the membrane (red). ***b*** Fractional current not inactivated 20 ms after depolarization start ($I_{20\,ms}$ / $I_{max}$) of the respective constructs before (white bars) and after hemin application (red bars). Data are means±SEM with *n* in parentheses.

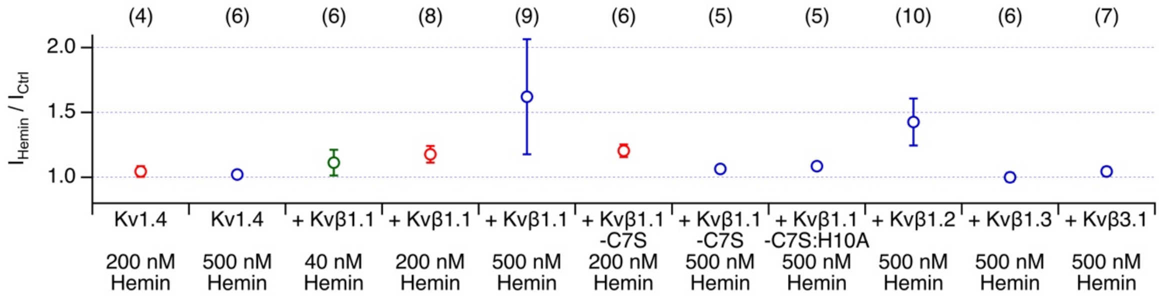


**Supplementary Figure 8. Peak current changes of Kv1.4 channels with coexpression of Kvβ subunits.**
Mean fractional change of the peak current of Kv1.4 channels or together with the Kvβ subunits in inside-out patches at 50 mV when subjected to hemin as indicated. Data are means±SEM with *n* in parentheses.

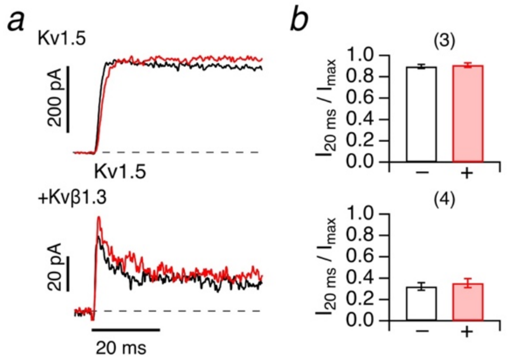


**Supplementary Figure 9. Kv1.5 and Kvβ1.3 subunits in *Xenopus* oocytes.**
Kv1.5 and Kvβ1.3 subunits in *Xenopus* oocytes. ***a*** Representative current traces at 40 mV from inside-out patches of *Xenopus* oocytes expressing Kv1.5 channels alone (*top*) or Kv1.5 plus Kvβ1.3 subunits before (black) and about 150 s after application of 200 nM hemin to the intracellular side of the membrane (red). ***b*** Fractional current not inactivated 20 ms after depolarization start ($I_{20\ ms} / I_{max}$) of the respective constructs before (white bars) and after hemin application (red bars). Data are means±SEM with *n* in parentheses.
